# PERSIST-PWI trial: Rationale and design of a multicenter randomized controlled trial comparing pulmonary vein isolation alone with pulmonary vein isolation plus posterior wall isolation using pulsed field ablation in patients with persistent atrial fibrillation

**DOI:** 10.1016/j.hroo.2026.03.013

**Published:** 2026-03-24

**Authors:** Yasuo Okumura, Michifumi Tokuda, Koichi Nagashima, Ryuta Watanabe, Yuji Wakamatsu, Shu Hirata, Masanaru Sawada, Hikaru Masuda, Kazuhiro Satomi, Masaomi Kimura, Koji Miyamoto, Hidehira Fukaya, Kenta Murotani, Kazuhiro Shimomura

**Affiliations:** 1 Division of Cardiology, Department of Medicine, Nihon University School of Medicine, Tokyo, Japan; 2 Division of Cardiology, Department of Internal Medicine, The Jikei University School of Medicine, Tokyo, Japan; 3 Department of Cardiology, Tokyo Medical University, Tokyo, Japan; 4 Department of Cardiology, Hirosaki University Graduate School of Medicine, Aomori, Japan; 5 Department of Cardiovascular Medicine, National Cerebral and Cardiovascular Center, Osaka, Japan; 6 Department of Cardiovascular Medicine, Kitasato University School of Medicine, Kanagawa, Japan; 7 Biostatistics Center, Kurume University, Fukuoka, Japan; 8 Department of Clinical Pharmacy, Aichi Cancer Center, Aichi, Japan

**Keywords:** Atrial fibrillation, Pulsed field ablation, Pulmonary vein isolation, Posterior wall isolation, Randomized controlled trial

## Abstract

**Background:**

Pulmonary vein isolation (PVI) is the cornerstone of atrial fibrillation (AF) ablation, yet outcomes remain suboptimal in persistent AF. With thermal energy, adjunctive left atrial posterior wall isolation (PWI) has shown inconsistent benefit, partly owing to limited lesion durability and esophageal safety constraints. Pulsed field ablation (PFA) may enable safer, more reproducible posterior wall lesion sets.

**Objective:**

This study aimed to compare PVI alone with PVI plus PWI using the FARAPULSE PFA system in symptomatic, drug-refractory persistent AF.

**Methods:**

PERSIST-PWI is an investigator-initiated, multicenter, open-label, parallel-group randomized controlled trial at approximately 60 sites in Japan. 500 patients with persistent AF (AF duration ≤3 years) will be randomized 1:1 to PVI alone or PVI + PWI. Pre- and postablation 3-dimensional mapping with OPAL HDx (FARAVIEW) is mandated. Follow-up includes visits at 3, 6, and 12 months with 12-lead electrocardiograms (ECGs), 24-hour Holter monitoring at 6 and 12 months, and biweekly plus symptom-triggered portable ECG transmissions.

**Results:**

The primary effectiveness endpoint is acute ablation success and freedom from clinically meaningful treatment failure from the end of an 8-week blanking period through 12 months, defined as documented AF/atrial flutter/atrial tachycardia (≥30 seconds on ambulatory monitoring or ≥10 seconds on 12-lead ECG), repeat ablation, electrical cardioversion, or initiation/escalation of class I/III antiarrhythmic drugs including amiodarone. Key secondary endpoints include AF burden, quality of life (Atrial Fibrillation Effect on Quality-of-Life questionnaire), and predefined safety events.

**Conclusion:**

PERSIST-PWI will provide randomized evidence on whether PFA-enabled PWI adds clinical benefit beyond PVI while maintaining safety in persistent AF, thereby informing contemporary ablation strategies.

## Graphical abstract

**Figure:**
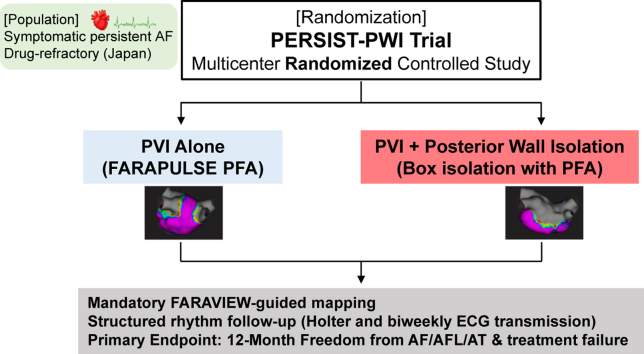

**Key Findings**

▪ In persistent atrial fibrillation (AF), routine posterior wall isolation (PWI) has shown inconsistent incremental benefit with thermal ablation, partly owing to limited lesion durability and esophageal safety constraints.
▪ Pulsed field ablation provides preferential myocardial injury with relative sparing of adjacent structures, potentially enabling safer and more durable posterior wall lesion sets.
▪ The PERSIST-PWI study is an investigator-initiated, multicenter randomized trial comparing pulmonary vein isolation (PVI) alone with PVI plus PWI using pulsed field ablation in symptomatic, drug-refractory persistent AF in Japan.
▪ Mandatory pre- and postablation FARAVIEW-guided mapping enables mechanistic assessment of lesion completeness and procedural reproducibility.
▪ Rhythm outcomes will be assessed using structured follow-up with Holter monitoring and biweekly or symptom-triggered portable electrocardiography transmissions.

## Introduction

Atrial fibrillation (AF) is an increasingly prevalent arrhythmia associated with stroke, heart failure, and impaired quality of life. Catheter ablation with pulmonary vein isolation (PVI) is highly effective for paroxysmal AF; however, in persistent AF, recurrence remains frequent even after apparently successful PVI,^1^^,^^2^ reflecting more advanced atrial remodeling and the presence of non–pulmonary vein (PV) substrates. Consequently, adjunctive strategies targeting additional atrial regions have been proposed,^3^, ^4^, ^5^ among which left atrial (LA) posterior wall isolation (PWI) has attracted particular interest because the posterior wall can participate in AF maintenance.^6^

Despite a compelling mechanistic rationale, randomized trials using thermal energy sources (radiofrequency or cryothermal) have not consistently demonstrated incremental benefit of routine PWI beyond PVI in persistent AF.^3^, ^4^, ^5^ This may be attributable to 2 major limitations: (1) incomplete or nondurable PV and posterior wall block owing to anatomic complexity and lesion discontinuity and (2) safety constraints, particularly the proximity of the posterior wall to the esophagus, which limits energy delivery and raises concern for collateral injury^7^^,^^8^—thereby restricting operators from creating sufficiently extensive and durable lesions in routine clinical practice.

Pulsed field ablation (PFA) is a nonthermal modality based on irreversible electroporation. It offers preferential myocardial injury with relative sparing of adjacent structures,^9^, ^10^, ^11^ potentially enabling wider lesion sets with improved safety margins. Contemporary data demonstrate high acute PVI success rates, low serious complication rates, and encouraging chronic durability of both PVI and PWI with PFA.^10^, ^11^, ^12^, ^13^ Accordingly, a key clinical question remains: do the technical and safety advantages of PFA translate into a clinically meaningful benefit of adding PWI to PVI in patients with persistent AF? This question cannot be answered without a dedicated randomized trial.

The PERSIST-PWI trial was designed to address this evidence gap by directly comparing PVI alone with PVI plus PWI performed with the FARAPULSE PFA system in Japanese patients with persistent AF.

## Methods

### Study design and population

PERSIST-PWI is an investigator-initiated, multicenter, randomized, open-label, parallel-group trial being conducted in Japan. Scientific oversight is provided by a physician-led steering committee. The steering committee consisted of Yasuo Okumura (Nihon University), Michifumi Tokuda (The Jikei University), Kazuhiro Satomi (Tokyo Medical University), Masaomi Kimura (Hirosaki University), Koji Miyamoto (National Cerebral and Cardiovascular Center), and Hidehira Fukaya (Kitasato University). The trial protocol was registered in the Japan Registry of Clinical Trials (jRCTs032250541; registered on December 1, 2025) and was approved by the central ethics committee at Nihon University Itabashi Hospital (clinical research judging committee; lead institution). In addition, study initiation at each participating site requires local approval by the site’s ethics committee and/or hospital director, as applicable (Supplemental Appendix). The research reported in this paper adheres to the ethical principles of the Declaration of Helsinki and applicable Japanese ethical and regulatory requirements and is designed and will be reported in accordance with the Consolidated Standards of Reporting Trials guidelines for randomized clinical trials. A written informed consent will be obtained from all participants before enrollment. Eligible patients will be randomized 1:1 to PVI alone (control) or PVI plus PWI (intervention) using the FARAPULSE PFA system.

### Trial oversight, event adjudication, and core laboratory

Scientific oversight is provided by a physician-led steering committee. Participant safety is overseen by an independent data and safety monitoring board, which periodically reviews unblinded safety data. Clinical events and key safety outcomes will be adjudicated by an independent clinical events committee (CEC) according to prespecified definitions.

To mitigate potential bias inherent to the open-label design, rhythm endpoints will be assessed by an independent arrhythmia core laboratory blinded to treatment assignment using electrocardiography (ECG)-based documentation collected per protocol (12-lead ECGs, Holter recordings, and portable ECG transmissions).

### Study population

Patients will be adults (≥18 years) with symptomatic persistent AF (continuous AF ≥7 days or requiring cardioversion) and an AF duration of ≤3 years, refractory or intolerant to at least 1 class I/III antiarrhythmic drug. The exclusion criteria include previous LA ablation (including PVI), LA diameter (LAD) of >5.5 cm, left ventricular ejection fraction of <40%, estimated glomerular filtration rate of <30 mL/min/1.73 m^2^, New York Heart Association functional class III/IV, major cardiovascular events within 90 days, secondary AF owing to reversible causes, conditions making LA instrumentation unsafe (eg, intracardiac thrombus), severe valvular disease or previous valve intervention, history of ventricular tachycardia/fibrillation, and contraindications to or inability to maintain anticoagulation (Table 1). The full inclusion and exclusion criteria are presented in Supplemental Table 1.

**Table 1:** Key inclusion and exclusion criteria

Inclusion criteria
1) Age ≥18 y
2) Symptomatic persistent AF (continuous AF ≥7 d or requiring cardioversion)
3) AF duration ≤3 y
4) Refractory or intolerant to ≥1 class I/III antiarrhythmic drug
5) Eligible for catheter ablation with PFA and able to provide a written informed consent
6) Willing and able to comply with protocol-specified follow-up and rhythm monitoring
Key exclusion criteria (as defined in protocol)
1) Previous left atrial ablation (including PVI)
2) Left atrial diameter >5.5 cm
3) Left ventricular ejection fraction <40%
4) eGFR <30 mL/min/1.73 m^2^
5) NYHA class III/IV heart failure
6) Major cardiovascular events within 90 d
7) Secondary AF owing to reversible causes
8) Intracardiac thrombus or other conditions making left atrial instrumentation unsafe
9) Severe valvular disease or previous valve intervention
10) History of ventricular tachycardia/fibrillation
11) Contraindication to, or inability to maintain, anticoagulation

Summary of inclusion and exclusion criteria. (Full criteria are presented in Supplemental Table 1).

AF = atrial fibrillation; eGFR = estimated glomerular filtration rate; NYHA = New York Heart Association; PFA = pulsed field ablation; PVI = pulmonary vein isolation.

### Randomization and stratification

**Figure 1:**
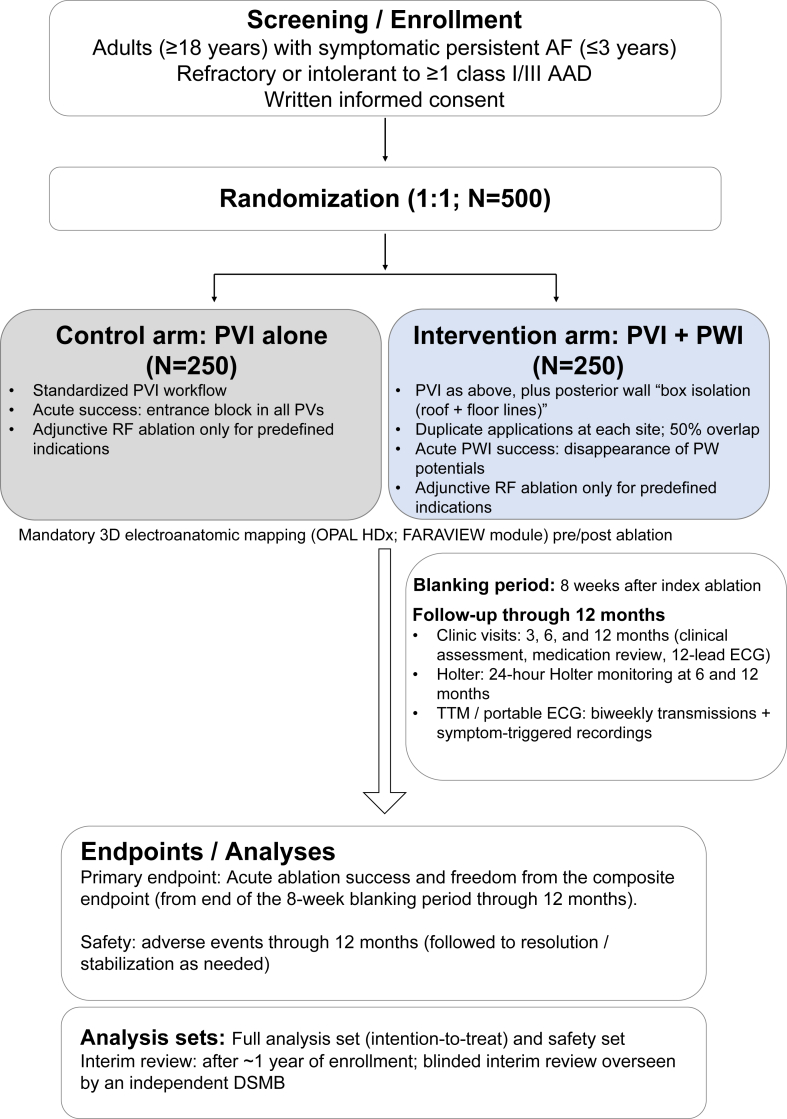
Overview of the PERSIST-PWI trial design. Adults with symptomatic, drug-refractory persistent AF (AF duration ≤3 years) are randomized 1:1 (N = 500) to PVI alone or PVI plus PWI using the FARAPULSE pulsed field ablation system. FARAVIEW-guided 3D mapping is performed before and after ablation. Rhythm follow-up includes clinic visits with 12-lead ECGs, scheduled Holter monitoring, and transtelephonic/portable ECG transmissions. The composite effectiveness endpoint is acute success and freedom from clinically meaningful treatment failure from the end of an 8-week blanking period to 12 months. 3D = 3-dimensional; AAD = antiarrhythmic drug; AF = atrial fibrillation; DSMB = data and safety monitoring board; ECG = electrocardiography; PV = pulmonary vein; PVI = pulmonary vein isolation; PW = posterior wall; PWI = posterior wall isolation; RF = radiofrequency; TTM = transtelephonic monitor.

Randomization will be performed centrally using a minimization-based dynamic allocation method. Patients will be allocated 1:1 to the PVI-alone group or the PVI + PWI group, stratified by site, age category (<75 vs ≥75 years), and LAD (<45 vs ≥45 mm). The overall study design is presented in Figure 1.

### Ablation procedures

All patients will undergo PVI using the FARAPULSE PFA system (Boston Scientific Japan K.K.). Acute PVI success will be defined as entrance block in all attempted PVs. As a rule, PVI will be performed using a standardized lesion delivery strategy (“two-pair basket plus two-pair flower” configuration per PV). During LA dwell, intravenous unfractionated heparin will be administered as needed to maintain an activated clotting time of >350 seconds.

3-dimensional electroanatomic mapping with the OPAL HDx mapping system (FARAVIEW software module) is mandatory to obtain LA and PV morphology. Pre- and postablation mapping will be performed using either the FARAWAVE 2.0 NAV PFA catheter or the IntellaMap Orion mapping catheter, according to operator preference and site availability. In sites where the IntellaMap Orion catheter is not available, pre-/postmapping with the FARAWAVE 2.0 NAV PFA catheter alone will be permitted. When the IntellaMap Orion catheter is used, LA access may be obtained via the FARADRIVE steerable sheath or another sheath sized ≥8.5F, with careful attention to air embolism, and the sheath will be exchanged to the FARADRIVE steerable sheath before insertion of the FARAWAVE 2.0 NAV PFA catheter for PVI, per protocol.

In the PVI + PWI group, additional posterior wall “box isolation” will be performed between the left and right PVs using anatomically guided applications with the FARAWAVE 2.0 NAV PFA catheter in the flower configuration, as described in the instructions for use. Roof and floor lines will be created (roof line along the inferior aspect of the superior PVs and floor line along the superior aspect of the inferior PVs). Particular attention will be paid to achieving complete cranial coverage of the LA roof to minimize the potential risk of roof-dependent macroreentrant atrial tachycardia (AT), with lesion completeness verified by FARAVIEW-guided mapping. Energy will be delivered at 2000 V with duplicate applications at each site and 50% overlap.^14^^,^^15^

In the PVI-alone group, only PVI will be performed and no additional posterior wall ablation will be undertaken. Catheter manipulation will be performed within the PV ostium and antrum, with care taken to avoid unintended energy delivery to the posterior wall.

Acute PVI success and PWI success will be confirmed by demonstration of entrance block with the disappearance of local PV and posterior wall field potentials. Where technically feasible, pacing from the PVs and posterior wall may be performed to assess exit block at the operator’s discretion.

No protocol-mandated waiting period is required, consistent with the pragmatic study design; reassessment after a short waiting period may be performed per operator discretion and site practice. Intracardiac echocardiography and/or fluoroscopic guidance may be used according to site practice and operator preference, and their use will be recorded. Field tag–based contact metrics are available at participating sites and may be incorporated into procedural assessment when feasible using a standardized approach informed by previous field tag–based analyses.^16^ In addition, emerging impedance-based contact sensing technologies may be incorporated as they become clinically available and will be analyzed exploratorily when applicable.

Adjunctive radiofrequency ablation (eg, cavotricuspid isthmus ablation) will be permitted in both groups only for predefined clinical indications, including documented non-PV triggers, sustained AT, typical atrial flutter, or other safety-driven needs; all adjunctive ablation will be recorded in the electronic data capture system. Procedural characteristics, including total procedure time (skin to skin), fluoroscopy time, acute PVI success, acute PWI success (PVI + PWI group only), and the presence and location of adjunctive ablation, will be collected.

### Follow-up and rhythm monitoring

Patient follow-up will consist of outpatient visits at 3, 6, and 12 months after the index ablation (day 0), including clinical assessment, review of medications, and 12-lead ECG at each visit. The use of antiarrhythmic drugs during follow-up will be systematically recorded in both treatment groups regardless of initiation or dose adjustment. Rhythm monitoring will include protocol-mandated 24-hour Holter monitoring at 6 and 12 months. In addition, transtelephonic monitoring and symptom-triggered recordings will be performed using an Omron portable ECG device (HCG-8060T), which will be provided by the study team. Participants will be instructed to transmit recordings biweekly after discharge and additionally at the time of symptoms suggestive of arrhythmia. Portable ECG transmissions are intended to complement intermittent Holter monitoring and improve capture of clinically relevant recurrences in real-world follow-up.

### Quality of life

Quality of life will be assessed using the Atrial Fibrillation Effect on Quality-of-Life questionnaire and the EQ-5D-5L at baseline and at 12 months.

### End of study and safety follow-up

The observation period is 12 months after ablation, and adverse events (AEs) will be collected from the procedure through 12 months, with additional follow-up as needed until recovery or stabilization. The schedule of study assessments is presented in Table 2.

**Table 2:** Schedule of study assessments

Observational items	Day −28 ∼ −1	Day 0	Day 1	3 mo ± 2 wk	6 mo ± 3 wk	12 mo ± 1 mo	Time of discontinuation	At the end of follow-up
Obtaining informed consent	○							
Background of study subjects	○							
Enrollment		○						
Physical examination and vital signs	○		○	○	○	○	○	○
Concomitant medications/therapies (including compliance information)	○	○	○	○	○	○	○	○
Assessment of symptoms (subjective symptoms)	○		○	○	○	○	○	○
ECG (12 leads)			○	○	○	○	○	○
24-h Holter monitoring (routine medical practice)					○	○		
TTM				Initiation∗	To continue	To continue		
Cardiac ultrasound (echocardiography)	○							
Blood tests (routine medical practice)	○		○					
Blood tests (hemolysis assessment)	○		○					
Urine tests (hemolysis assessment)	○		○					
Ablation procedure information		○						
Evaluation of adverse events/complications			○	○	○	○	○	○
Symptomatology and QOL assessment (AFEQT, EQ-5D-5L)	○					○		
Presence of recurrent AF (subjective/objective)			○	○	○	○		

Schedule of protocol-specified visits and assessments from screening through 12-month follow-up, including ECG-based rhythm surveillance, Holter monitoring, and transtelephonic/portable ECG transmissions.

AF = atrial fibrillation; AFEQT = Atrial Fibrillation Effect on Quality-of-Life; ECG = electrocardiography; QOL =quality of life; TTM = transtelephonic monitor.

∗ Visit should be 3 months to match the routine clinical visit. However, the usage of TTM shall be explained at the time of discharge from the hospital, and measurements will begin the day after discharge.

### Endpoints

The primary composite effectiveness endpoint, aligned with previous PFA trials (eg, ADVANTAGE AF^14^^,^^15^), is defined as acute ablation success and freedom from clinically meaningful treatment failure during the postblanking monitoring period (from the end of an 8-week blanking period to 12 months). Treatment failure is defined as a composite of documented AF/atrial flutter/AT (≥30 seconds on ambulatory monitoring or ≥10 seconds on 12-lead ECG), repeat ablation, electrical cardioversion, or initiation/escalation of class I/III antiarrhythmic drugs including amiodarone. Secondary endpoints include the individual components of the primary endpoint, time to documented AF recurrence, AF burden based on rhythm-monitoring data, change in quality of life (Atrial Fibrillation Effect on Quality-of-Life and EQ-5D-5L), and predefined safety outcomes including stroke or transient ischemic attack, cardiac tamponade/pericarditis, major vascular complications, and major bleeding. Phrenic nerve injury (symptomatic or asymptomatic) will also be prospectively collected and adjudicated as a prespecified safety outcome. Additional late safety surveillance (eg, PV stenosis and atrioesophageal fistula) will be captured as prespecified AEs and serious AEs. Exploratory analyses will evaluate posterior wall block durability in relation to recurrence, trajectories of hemolysis biomarkers and renal function, and prespecified subgroup effects (Table 3). Full endpoint definitions and ascertainment are presented in Supplemental Table 2. In cases of repeat ablation, remapping data (PV reconnection and posterior wall conduction status, when assessed) will be captured in the electronic data capture and analyzed exploratorily.

**Table 3:** Primary, secondary, and exploratory endpoints

Category	Endpoints
Primary effectiveness endpoints	Acute ablation success and freedom from the following during the postblanking monitoring period (from the end of an 8-wk blanking period through 12 mo), defined as a composite of: 1) Documented AF/AFL/AT (≥30 s on ambulatory monitoring or ≥10 s on 12-lead ECG) 2) Repeat ablation for AF/AFL/AT 3) Electrical cardioversion for AF/AFL/AT 4) Initiation/escalation of class I/III antiarrhythmic drugs including amiodarone
Key secondary endpoints	1) Individual components of the primary composite endpoint 2) Time to documented AF recurrence 3) AF burden based on rhythm-monitoring data 4) Change in quality of life: AFEQT and EQ-5D-5L 5) Safety outcomes (see Supplemental Table 2 for definitions)
Exploratory endpoints	1) Association between posterior wall block durability and recurrence 2) Trajectories of hemolysis biomarkers and renal function 3) Prespecified subgroup effects (age, sex, BMI, LAD, AF duration, DR-FLASH score)

Overview of study endpoints. Detailed endpoint definitions and ascertainment procedures are presented in Supplemental Table 2.

AF = atrial fibrillation; AFEQT = Atrial Fibrillation Effect on Quality-of-Life; AFL = atrial flutter; AT = atrial tachycardia; BMI = body mass index; ECG = electrocardiography; LAD = left atrial diameter.

### Statistical considerations

Sample size was informed by previous randomized trials of PWI using thermal energy sources (eg, STAR AF II,^3^ CAPLA,^4^ and other studies^5^^,^^17^^,^^18^), in which the 1-year freedom from atrial arrhythmia after PVI alone ranged from approximately 53% to 71% and incremental benefit of routine PWI was not consistently demonstrated. In contrast, contemporary PFA technology may enable more durable and safer posterior wall lesion sets, which could translate into improved clinical effectiveness.

Based on published PFA outcomes and supporting prospective data,^14^^,^^15^ the assumed 12-month freedom from the primary composite effectiveness endpoint was set at 60% for the PVI-alone group and 72.5% for the PVI + PWI group (absolute difference 12.5%). With a 2-sided type I error probability of .05 and 80% power, 224 participants per group (total 448) are required. To account for an anticipated 10% dropout rate, the planned enrollment is 250 participants per group, resulting in a total sample size of 500.

Effectiveness analyses will be conducted according to the intention-to-treat principle. Safety analyses will be performed in all participants who received study treatment (safety set). For the primary composite effectiveness endpoint, time-to-event analyses will focus on the postblanking treatment failure component; acute ablation success will be summarized descriptively by group. Time-to-event outcomes will be analyzed using Kaplan–Meier methods and compared with the log-rank test; hazard ratios and 95% confidence intervals will be estimated using Cox proportional hazards models, with prespecified covariate adjustment as appropriate. Rhythm endpoint documentation will be reviewed by the blinded arrhythmia core laboratory, and major safety outcomes will be adjudicated by the independent CEC. Prespecified subgroup analyses (age, sex, body mass index, LAD, AF duration, DR-FLASH score^19^) will be assessed using treatment × subgroup interaction terms. Missing data will be handled primarily by complete-case analysis, with multiple imputation and sensitivity analyses considered if missingness is substantial, as specified in the statistical analysis plan. A single interim review is planned after approximately 1 year of enrollment and will be conducted blinded under the oversight of an independent data and safety monitoring board. No formal interim hypothesis testing for effectiveness is planned. Analyses will be performed using SAS software (version 9.4 or later, SAS Institute Inc, Cary, NC).

## Discussion

The PERSIST-PWI trial was specifically designed to test this hypothesis in a rigorous and pragmatic manner. By directly comparing PVI alone with PVI plus PWI using a standardized PFA workflow, the trial isolates the incremental effect of PWI under conditions where durable lesion creation and safety margins are expected to be optimized. Importantly, the trial incorporates mandatory pre- and postablation 3-dimensional electroanatomic mapping using FARAVIEW-guided OPAL HDx technology, enabling mechanistic assessment of PWI durability and its relationship to clinical recurrence—an aspect largely absent from previous randomized studies. This trial complements previous PFA trials by evaluating generalizability and mechanistic durability in a Japanese multicenter setting with mandatory FARAVIEW-guided mapping.

Another strength of PERSIST-PWI is its structured rhythm-monitoring strategy, which combines scheduled Holter monitoring with biweekly and symptom-triggered portable ECG transmissions. This approach aims to balance feasibility in a large, multicenter investigator-initiated trial with enhanced detection of clinically meaningful arrhythmia recurrence, thereby improving the robustness of effectiveness assessment in real-world follow-up.

In addition, a key design feature of PERSIST-PWI is the deliberate harmonization of outcome definitions and rhythm-monitoring intensity with contemporary FARAPULSE PFA trials, particularly ADVANTAGE AF.^14^^,^^15^ By adopting a comparable primary composite effectiveness endpoint and a similarly structured follow-up strategy, this trial was intentionally designed to facilitate cross-trial contextual interpretation of clinical effectiveness and safety in patients undergoing PFA for persistent AF. This harmonization was prespecified to enhance interpretability against a rapidly evolving PFA evidence base while allowing PERSIST-PWI to address a distinct mechanistic question—namely, whether PWI provides incremental benefit beyond PVI when performed using contemporary PFA technology.

Several limitations should be acknowledged. The open-label design is inherent to interventional ablation trials; however, this is mitigated by independent adjudication of clinical events by a CEC and blinded rhythm assessment by an arrhythmia core laboratory. Continuous rhythm monitoring with an implantable loop recorder was not used; therefore, AF burden estimates may be less precise than those obtained with continuous monitoring, and asymptomatic episodes may be underdetected despite frequent portable ECG transmissions. In addition, although the trial is conducted exclusively in Japan, the procedural workflow, energy modality, and endpoint definitions are aligned with contemporary international PFA trials, supporting broader interpretability of the findings.

## Conclusion

PERSIST-PWI is a large-scale, investigator-initiated randomized trial designed to address a central unresolved question in persistent AF ablation: whether routine PWI confers incremental clinical benefit when performed using contemporary PFA technology. By integrating standardized PFA workflows, rigorous safety oversight, comprehensive rhythm monitoring, and mechanistic mapping assessment, this trial is positioned to define the clinical and procedural value of adding PWI to PVI in persistent AF. The results of PERSIST-PWI are expected to inform future ablation strategies and procedural standards in the evolving era of nonthermal AF ablation.

## Disclosures

Dr Okumura reports honoraria from AstraZeneca and Johnson & Johnson; honoraria from Bristol Myers Squibb, Medtronic, Otsuka Pharmaceutical, Boston Scientific, Daiichi Sankyo, and Bayer; research funding from Biosense Webster; and endowed chair support from Abbott, Japan Lifeline, Medtronic, Boston Scientific, and Biotronik. Dr Nagashima reports honoraria from Johnson & Johnson, Medtronic, and Boston Scientific. Dr Satomi reports honoraria from Boston Scientific, Japan Lifeline, Medtronic, Abbott, and Daiichi Sankyo and research funding from Biotronik. Dr Tokuda reports honoraria from Medtronic and research funding from Japan Lifeline. Dr Kimura is an associate professor in the Department of Advanced Management of Cardiac Arrhythmias, an endowment department supported by Medtronic and Japan Lifeline, and reports honoraria from Medtronic, Boston Scientific, Johnson & Johnson, and Toray. Dr Miyamoto reports funding/grants from Medtronic, Biosense Webster, Abbott, and Boston Scientific and honoraria from Medtronic, Biosense Webster, Abbott, and Boston Scientific, all outside the submitted work, and is also affiliated with a department endowed by Medtronic outside the submitted work. Dr Fukaya reports honoraria from Medtronic and Daiichi Sankyo and honoraria from Abbott. Murotani reports honoraria from Chugai Pharmaceutical, AstraZeneca, and Taiho Pharmaceutical.

## Footnotes

Appendix Supplementary data associated with this article can be found in the online version at https://doi.org/10.1016/j.hroo.2026.03.013.
