# Supplementary material for: PERSIST-PWI trial: Rationale and design of a multicenter randomized controlled trial comparing pulmonary vein isolation alone with pulmonary vein isolation plus posterior wall isolation using pulsed field ablation in patients with persistent atrial fibrillation
Source: Heart Rhythm O2. 2026 Mar 24;7(6):1182–9. doi: 10.1016/j.hroo.2026.03.013 (PMC13307492; doi:10.1016/j.hroo.2026.03.013)
Supplement: Supplemental Material [file mmc1.docx]

**Supplemental Appendix**

| Steering Committee |
| --- |
| Yasuo Okumura (Nihon University School of Medicine, Tokyo, Japan) Michifumi Tokuda (The Jikei University School of Medicine, Tokyo, Japan) Kazuhiro Satomi (Tokyo Medical University, Tokyo, Japan) Masaomi Kimura (Hirosaki University Graduate School of Medicine, Aomori, Japan) Koji Miyamoto (National Cerebral and Cardiovascular Center, Osaka, Japan) Hidehira Fukaya (Kitasato University School of Medicine, Kanagawa, Japan) |
| Independent Data and Safety Monitoring Board (DSMB) |
| Kazuo Matsumoto (Saitama Medical University International Medical Center, Saitama, Japan)  Yoshinori Kobayashi (Tokai University Hachioji Hospital, Tokyo, Japan)  The DSMB is independent of the investigators and sponsor. |
| List of participating centers (66 centers) |
| 1. Nihon University School of Medicine, Tokyo, Japan 2. The Jikei University School of Medicine, Tokyo, Japan 3. Tokyo Medical University, Tokyo, Japan 4. Hirosaki University Graduate School of Medicine, Aomori, Japan 5. National Cerebral and Cardiovascular Center, Osaka, Japan 6. Kitasato University School of Medicine, Kanagawa, Japan 7. Yokkaichi Municipal Hospital, Mie, Japan 8. Shonan Kamakura General Hospital, Kanagawa, Japan 9. Yamagata University Hospital, Yamagata, Japan 10. Saiseikai Fukuoka General Hospital, Fukuoka, Japan 11. Kawasaki Saiwai Hospital, Kanagawa, Japan 12. Kanazawa University Hospital, Ishikawa, Japan 13. Kyoto University Hospital, Kyoto, Japan 14. Yamaguchi University Hospital, Yamaguchi, Japan 15. Tsuchiura Kyodo General Hospital, Ibaraki, Japan 16. Japanese Red Cross Nagoya Daini Hospital, Aichi, Japan 17. Mitsubishi Kyoto Hospital, Kyoto, Japan 18. Oita University Hospital, Oita, Japan 19. Gunma Cardiovascular Center, Gunma, Japan 20. National Hospital Organization Osaka National Hospital, Osaka, Japan 21. Sendai Kousei Hospital, Miyagi, Japan 22. Hamamatsu University Hospital, Shizuoka, Japan 23. Institute of Science Tokyo Hospital, Tokyo, Japan 24. Hiroshima University Hospital, Hiroshima, Japan 25. Toho University Sakura Hospital, Chiba, Japan 26. Tokai University Hospital, Kanagawa, Japan 27. Toyota Memorial Hospital, Aichi, Japan 28. Kyoto-Katsura Hospital, Kyoto, Japan 29. Kyorin University Hospital, Tokyo, Japan 30. Jichi Medical University Hospital, Tochigi, Japan 31. Iwate Medical University Hospital, Iwate, Japan 32. Hyogo Prefectural Harima-Himeji General Medical Center, Hyogo, Japan 33. Keio University Hospital, Tokyo, Japan 34. Hiroshima Prefectural Hospital, Hiroshima, Japan 35. Miyazaki Medical Association Hospital, Miyazaki, Japan 36. Kameda Medical Center, Chiba, Japan 37. Komaki City Hospital, Aichi, Japan 38. Tosei General Hospital, Aichi, Japan 39. Sakakibara Heart Institute, Tokyo, Japan 40. Nagoya University Hospital, Aichi, Japan 41. Juntendo University Hospital, Tokyo, Japan 42. Japanese Red Cross Wakayama Medical Center, Wakayama, Japan 43. Hiroshima City Hiroshima Citizens Hospital, Hiroshima, Japan 44. Ageo Central General Hospital, Saitama, Japan 45. Dokkyo Medical University Saitama Medical Center, Saitama, Japan 46. Gunma University Hospital, Gunma, Japan 47. Osaka Keisatsu Hospital, Osaka, Japan 48. Kindai University Hospital, Osaka, Japan 49. Fujita Health University Hospital, Aichi, Japan 50. Showa Medical University Fujigaoka Hospital, Kanagawa, Japan 51. Kobe University Hospital, Hyogo, Japan 52. Shizuoka City Shizuoka Hospital, Shizuoka, Japan 53. Medical Corporation Sapporo Heart Center, Sapporo Cardiovascular Clinic, Hokkaido, Japan 54. Juntendo University Urayasu Hospital, Chiba, Japan 55. University of Fukui Hospital, Fukui, Japan 56. Kurume University Hospital, Fukuoka, Japan 57. Ogaki Municipal Hospital, Gifu, Japan 58. Dokkyo Medical University Hospital, Tochigi, Japan 59. Tokyo Metropolitan Hiroo Hospital, Tokyo, Japan 60. Chiba University Hospital, Chiba, Japan 61. Kyoto Prefectural University of Medicine Hospital, Kyoto, Japan 62. Kokura Memorial Hospital, Fukuoka, Japan 63. Yokohama Minato Red Cross Hospital, Kanagawa, Japan 64. Shiga General Hospital, Shiga, Japan 65. Takai Hospital, Osaka, Japan 66. Takeda Hospital, Kyoto, Japan |
