# Supplementary material for: PERSIST-PWI trial: Rationale and design of a multicenter randomized controlled trial comparing pulmonary vein isolation alone with pulmonary vein isolation plus posterior wall isolation using pulsed field ablation in patients with persistent atrial fibrillation
Source: Heart Rhythm O2. 2026 Mar 24;7(6):1182–9. doi: 10.1016/j.hroo.2026.03.013 (PMC13307492; doi:10.1016/j.hroo.2026.03.013)
Supplement: Supplemental Table 1 [file mmc2.docx]

**Supplemental Table S1. Full inclusion and exclusion criteria**

| Inclusion criteria |
| --- |
| Patients meeting all of the following criteria will be eligible:   1. Age ≥18 years at the time of written informed consent. 2. Persistent AF, defined as continuous AF for ≥7 days or requiring cardioversion, documented by 12-lead ECG or Holter monitoring. 3. Symptomatic persistent AF that is refractory to, intolerant of, or contraindicated for at least 1 class I or III antiarrhythmic drug. 4. Duration of AF ≤3 years. 5. Willing and able to comply with all follow-up assessments and testing at the participating study center. 6. Provided written informed consent.   Rationale (inclusion): Criteria were selected to enroll a representative population with symptomatic, drug-refractory persistent AF suitable for evaluating the comparative effectiveness and safety of the two ablation strategies. |
| Exclusion criteria |
| Patients meeting any of the following criteria will be excluded:   1. Atrial, valvular, and myocardial conditions 2. Prior left atrial ablation (including PVI). 3. Left atrial diameter (LAD) >55 mm on echocardiography performed within 4 weeks prior to informed consent. 4. Left ventricular ejection fraction (LVEF) <40% on echocardiography performed within 4 weeks prior to informed consent. 5. Conditions in which catheter insertion/manipulation in the cardiac chambers is unsafe (e.g., intracardiac thrombus or myxoma; recent cardiac surgery with atriotomy). 6. Prior heart valve intervention (replacement, ring, repair, balloon valvuloplasty, etc.). 7. Severe valvular disease (symptomatic, worsening heart failure, or associated with abnormal LV function/hemodynamics). 8. Hypertrophic cardiomyopathy. 9. Congenital heart disease or major rotational anomalies that increase procedural risk. 10. Arrhythmia/device related     1. History of ventricular tachycardia or ventricular fibrillation.     2. Secondary AF due to reversible or non-cardiac causes (e.g., electrolyte imbalance, thyroid disease, alcohol).     3. Continuous atrial pacing devices (e.g., pacemaker/ICD) that could confound rhythm assessment after ablation.     4. Intra-atrial baffles or foramen ovale patches. 11. Recent major surgery/event (within the past 90 days) 12. Myocardial infarction, unstable angina, or coronary intervention. 13. Cardiac surgery. 14. Heart failure hospitalization. 15. Pericarditis or symptomatic pericardial effusion. 16. Gastrointestinal bleeding. 17. Stroke, transient ischemic attack (TIA), or intracranial bleeding. 18. Non-central thromboembolism. 19. Carotid stenting or endarterectomy. 20. Infection, pulmonary, gastrointestinal, and renal conditions 21. Active systemic infection. 22. Severe lung disease, pulmonary hypertension, abnormal blood gas findings, or respiratory disease requiring supplemental oxygen. 23. Renal dysfunction (eGFR <30 mL/min/1.73 m²), history of dialysis, or renal transplant. 24. Clinically significant esophageal/gastric disorders (e.g., severe/erosive esophagitis, uncontrolled reflux, gastroparesis, esophageal candidiasis, active gastroduodenal ulceration). 25. History or evidence of diaphragmatic paralysis/paresis. 26. Other diseases and conditions     1. NYHA class III or IV heart failure.     2. Uncontrolled hypertension (SBP >160 mmHg or DBP >95 mmHg on two baseline measurements).     3. Bleeding disorders or inability to maintain adequate anticoagulation.     4. Active hematologic disorders (e.g., bleeding diathesis, thrombocytopenia, leukemia, malignant lymphoma, myelodysplastic syndrome).     5. Vena cava embolic protection filter and/or known femoral thrombus requiring femoral approach.     6. History of organ or hematopoietic stem cell transplantation or undergoing evaluation for transplantation.     7. Active or ongoing malignancy (remission permitted).     8. Severe obesity (BMI >40.0 kg/m²).     9. Uncontrolled diabetes mellitus or HbA1c >8.0% documented within 90 days prior to consent.     10. Untreated severe obstructive sleep apnea (apnea–hypopnea index >30).     11. Contraindication to anticoagulation or inability/unwillingness to use anticoagulation.     12. Not on anticoagulation therapy for ≥4 weeks prior to the ablation procedure.     13. Predicted life expectancy <1 year. 27. Women of childbearing potential who are pregnant, lactating, or have no adequate birth control 28. Patients who are currently enrolled in another investigational study or registry that would interfere with the current study (excluding governmental registry or a purely observational study without treatment). 29. Patients judged as inappropriate by the principal investigator or subinvestigator in terms of safety or eligibility   Rationale (exclusion): Criteria were selected to minimize confounding, ensure participant safety, and preserve interpretability of effectiveness and safety outcomes. |
