# Supplementary material for: PERSIST-PWI trial: Rationale and design of a multicenter randomized controlled trial comparing pulmonary vein isolation alone with pulmonary vein isolation plus posterior wall isolation using pulsed field ablation in patients with persistent atrial fibrillation
Source: Heart Rhythm O2. 2026 Mar 24;7(6):1182–9. doi: 10.1016/j.hroo.2026.03.013 (PMC13307492; doi:10.1016/j.hroo.2026.03.013)
Supplement: Supplemental Table 2 [file mmc3.docx]

**Supplemental Table S2. Endpoint definitions and ascertainment**

| **Endpoint** | **Definition** | **Time window** | **Ascertainment / Data sources** |
| --- | --- | --- | --- |
| **Acute PVI success** | Entrance block in all targeted pulmonary veins at the end of the index procedure (per protocol). | Index procedure | Procedural records; mapping findings recorded in the EDC. |
| **Acute PWI success (PVI+PWI group)** | Disappearance of local posterior wall potentials within the posterior wall “box” (per protocol-defined criteria). | Index procedure | Procedural records; mapping findings recorded in the EDC. |
| **Primary composite effectiveness endpoint (postblanking freedom from events)** | **Acute ablation success and freedom from the following events during the postblanking monitoring period:** (1) documented AF/AFL/AT meeting ECG criteria; (2) repeat ablation for AF/AFL/AT; (3) any electrical cardioversion for AF/AFL/AT; (4) initiation/escalation of class I/III AADs including amiodarone. | From end of 8-week blanking through 12 months | (1) Rhythm documentation: 12-lead ECGs at follow-up visits, Holter monitoring, and TTM/portable ECG transmissions; (2) Procedures/medication changes: medical records and EDC. Time-to-first event will be analyzed from the end of the blanking period through 12 months using Kaplan–Meier methods and Cox proportional hazards models, as prespecified in the SAP. |
| **Documented AF/AFL/AT** | AF/AFL/AT documented by: ≥30 seconds on ambulatory monitoring (Holter or TTM/portable ECG), or ≥10 seconds on 12-lead ECG. | From end of 8-week blanking through 12 months | ECG tracings, Holter reports, and transmitted recordings uploaded/archived per site practice and recorded in the EDC. |
| **Repeat ablation** | Any repeat catheter ablation performed for AF/AFL/AT after the index procedure. | From end of 8-week blanking through 12 months | Medical records/EDC. |
| **Electrical cardioversion** | Any electrical cardioversion performed for AF/AFL/AT after the index procedure. | From end of 8-week blanking through 12 months | Medical records/EDC. |
| **Initiation/escalation of AADs** | Initiation or dose escalation of class I/III AADs, including amiodarone, after the postblanking period (protocol-defined capture rules). | From end of 8-week blanking through 12 months | Concomitant medication logs and medical records; recorded in the EDC. |
| **Time to documented AF recurrence** | Time to first documented AF recurrence meeting ECG criteria (AF only; excluding AFL/AT if prespecified). | From end of 8-week blanking through 12 months | Same rhythm data sources as above. |
| **AF burden** | Proportion of monitoring time spent in AF/AFL/AT based on available rhythm-monitoring data; calculated using prespecified rules in the statistical analysis plan (SAP). | From discharge through 12 months (analysis window per SAP) | Holter monitoring at 6 and 12 months (routine practice) plus transmitted recordings (TTM/portable ECG); calculation rules specified in the SAP. |
| **Quality of life (AFEQT, EQ-5D-5L)** | Change from baseline to 12 months in AFEQT and EQ-5D-5L scores (protocol-specified scoring). | Baseline and 12 months | Patient-reported questionnaires collected per schedule and recorded in the EDC. |
| **Safety event collection (overall)** | All adverse events (AEs) and serious adverse events (SAEs) captured and categorized using protocol-defined criteria. | Index procedure through 12 months (with additional follow-up until resolution/stabilization as needed) | Site reporting at each visit and interim contacts; source documents and EDC entry; SAE reporting per protocol. |
| **Stroke** | Acute focal neurological deficit consistent with stroke, with imaging confirmation when available and/or persistent deficit per standard clinical definitions. | Index procedure through 12 months | Clinical assessment, imaging reports, discharge summaries; recorded in EDC. |
| **TIA** | Transient neurological deficit without infarction on imaging when available, per protocol definition. | Index procedure through 12 months | Clinical assessment and imaging when available; recorded in EDC. |
| **Cardiac tamponade** | Pericardial effusion with hemodynamic compromise requiring pericardiocentesis and/or surgical intervention. | Index procedure through 12 months | Procedural/clinical records; echocardiography reports when available; recorded in EDC. |
| **Pericarditis** | Clinically diagnosed per protocol (symptoms and supportive findings such as ECG changes, biomarkers, and/or imaging when available). | Index procedure through 12 months | Clinical records; ECG/labs/imaging if performed; recorded in EDC. |
| **Major vascular complication** | Access-site complication requiring transfusion, surgical/endovascular intervention, or prolonging hospitalization (per protocol-defined threshold). | Index procedure through 12 months | Clinical records; procedure notes; recorded in EDC. |
| **Major bleeding** | Major bleeding defined by protocol (e.g., ISTH major bleeding or protocol-specified definition). | Index procedure through 12 months | Clinical records, labs, transfusion records; recorded in EDC. |
| **Phrenic nerve injury (PNI)** | Phrenic nerve injury detected during or after the index procedure, including symptomatic or asymptomatic diaphragmatic dysfunction, as documented by procedural testing and/or post-procedural clinical assessment and imaging when available. Recovery status (complete/partial/no recovery) will be recorded. | Index procedure through 12 months | Procedural records (including phrenic nerve monitoring/testing where performed), clinical assessment at follow-up visits, imaging (e.g., chest radiography/ultrasound/fluoroscopy) when clinically indicated; recorded in the EDC. |
| **Pulmonary vein (PV) stenosis** | Imaging-confirmed clinically significant PV stenosis (per protocol definition). | Index procedure through 12 months | Imaging reports when performed clinically; recorded in EDC. |
| **Atrioesophageal fistula (AEF)** | Clinically suspected or confirmed AEF per standard clinical criteria (rare event; monitored throughout follow-up). | Index procedure through 12 months | Clinical diagnosis/imaging; immediate SAE reporting; recorded in EDC. |
| **Hemolysis-associated acute kidney injury (exploratory safety signal)** | AKI temporally associated with hemolysis (definition and thresholds prespecified in the SAP/protocol), assessed alongside hemolysis biomarkers and renal function. | Per protocol schedule through 12 months | Routine labs and protocol-specified hemolysis/renal assessments; recorded in EDC; analyzed exploratorily. |
| **Hemolysis biomarkers / renal function trajectories (exploratory)** | Trajectories of hemolysis biomarkers (e.g., LDH, haptoglobin, plasma free hemoglobin) and renal function (serum creatinine/eGFR) per schedule; exploratory analyses per SAP. | Per protocol schedule through 12 months | Blood/urine tests per schedule; recorded in EDC. |
| **Event oversight / interim review** | Safety trends reviewed under independent DSMB governance; interim review conducted blinded per charter/protocol. | During enrollment and follow-up | DSMB reports/meeting minutes per governance process (not part of endpoint ascertainment but oversight). |
